# Simultaneous Detection and Differentiation of Human Papillomavirus Genotypes 6, 11, 16 and 18 by AllGlo Quadruplex Quantitative PCR

**DOI:** 10.1371/journal.pone.0048972

**Published:** 2012-11-09

**Authors:** Daojun Yu, Yu Chen, Shenghai Wu, Baohong Wang, Yi-Wei Tang, Lanjuan Li

**Affiliations:** 1 State Key Laboratory for the Diagnosis and Treatment of Infectious Diseases, The First Affiliated Hospital, School of Medicine, Zhejiang University, Hangzhou, China; 2 Department of Clinical Laboratories, Hangzhou First People’s Hospital, Hangzhou, China; 3 Clinical Microbiology Service, Memorial Sloan-Kettering Cancer Center, New York, New York, United States of America; University of Houston, United States of America

## Abstract

**Background:**

Human papillomaviruses (HPV) are classified into high-risk HPV and low-risk HPV. The most common high-risk HPV types in cervical cancer are HPV 16 and 18, and the most common low-risk types causing genital warts are HPV 6 and HPV 11. In this study, applying novel AllGlo fluorescent probes, we established a quadruplex quantitative PCR method to simultaneously detect and differentiate HPV 6, 11, 16 and 18 in a single tube.

**Methods:**

The specificity, the sensitivity, the detection limit, the reproducibility and the standard curve of this method were examined. Finally, clinical samples that had been tested previously by TaqMan PCR and HPV GenoArray (GA) test were used to verify the accuracy and sensitivity of the method.

**Results:**

The assay has a sensitivity of 10^1^ to 10^2^ copies/test and a linear detection range from 10^1^ to 10^8^ copies/test. The mean amplification efficiencies for HPV 6, 11, 16, and 18 were 0.97, 1.10, 0.93 and 1.20, respectively, and the mean correlation coefficient (r^2^) of each standard curve was above 0.99 for plasmid templates ranging from 10^3^ to 10^7^ copies/test. There was 100% agreement between the AllGlo quadruplex quantitative PCR, HPV GA test and TaqMan uniplex qPCR methods.

**Conclusions:**

AllGlo quadruplex quantitative PCR in a single tube has the advantages of relatively high throughput, good reproducibility, high sensitivity, high specificity, and a wide linear range of detection. The convenient single tube format makes this assay a powerful tool for the studies of mixed infections by multiple pathogens, viral typing and viral load quantification.

## Introduction

More than 120 Human papillomaviruses (HPV) genotypes have been found throughout the world. The most common high-risk HPV types in cervical cancer are HPV 16 and 18, and the most common low-risk types causing genital warts are HPV 6 and HPV 11 [1]. Some studies suggest that about 70% of cervical epithelial cell dysplasia and cervical cancer is closely related to HPV 16 and 18 infection, while more than 90% of external genital warts is caused by HPV 6 and 11 infection [2], [3]. HPV 16 and 18 are regarded as high-risk HPV, while HPV 6 and 11 are thought as low-risk HPV [4]–[6]. A persistent infection may generate a particularly high HPV DNA load through viral replication [7]–[9]. Because HPV 6 or 11 can cause genital warts and HPV 16 or 18 may lead cervical cancer, preventing infections from these viruses can be an effective way to control the incidence of cervical cancer and cauliflower excrescence. A natural HPV-infection gives little or none protection against new infections due to low immune response, but the HPV-vaccine does.Several HPV vaccines are commercially available [10]–[16] and have been shown to be effective in preventing infections caused by HPV 6, 11, 16 and 18, There are already several commercial tests for HPV 6, 11, 16 and 18 available. It is obvious a marked progression for these tests. This assay will facilitate molecular epidemiological studies and drug development.

HPV genotyping is mainly determined by molecular methods of detecting viral DNA [17]–[21]. The multiplex real-time fluorescence quantitative PCR (qPCR) technique is an effective method which can simultaneously type many viruses [22–24]. However, existing qPCR has some disadvantages such as low throughput [25,26], complicated techniques [27,28], and expensive reagents [27,29]. Moreover, it was not able to type four HPV viruses simultaneously in a single tube in a quantitative manner [26] in order to meet the requirement for detecting multiple subtypes of HPV in a clinical setting.

**Figure 1:**
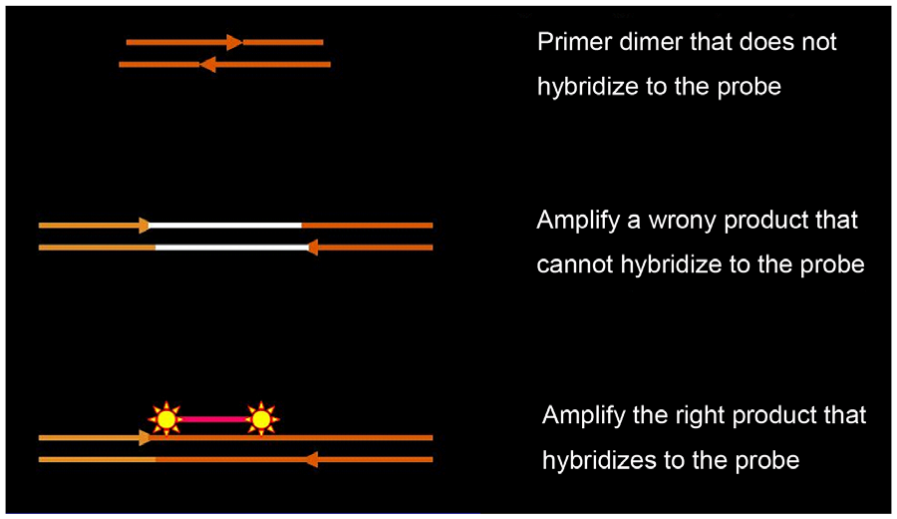
AllGlo probe fluorogenic detection systems for quantitative PCR. The advantage of AllGlo probe is brighter than TaqMan, no inhibition, higher specificity than TaqMan, easier multiplexing probe design.

The AllGlo probe is the latest generation of fluorescent quantitative probes developed by AlleLogic Biosciences Corporation (Hayward, California). It replaces the quencher with a fluorophore that is identical the first fluorophore. Both of the fluorophores are in a quenched state when the probe is intact, but become dequenched when the probe is cleaved. An AllGlo probe generates two light-emitting fluorophores per cleavage event, leading to greater fluorescence gain in fluorescence PCR and lower Ct value (Figure 1). In addition, the optimal Tm of the oligonucleotide of the AllGlo probe is the same temperature for annealing/extension, not ten degrees higher as is required for TaqMan. As a result, AllGlo probes tend to be shorter, usually 15–16 nucleotides long. This short oligonucleotide is advantageous in single mismatch discrimination. So AllGlo quadruplex quantitative PCR has the advantages of relatively high throughput, good reproducibility, high sensitivity, high specificity, it is easy for designing the probes and primers of multiplex qPCR and can increase the detection throughput.

In this study, we used AllGlo probe and developed a method to simultaneously detect four types of HPV (HPV 6, 11, 16, 18) in a single-tube format. This quadruplex assay was validated with an existing uniplex TaqMan assay and a HPV GA test. We carried out a series of control experiments and demonstrated the high specificity, sensitivity, and repeatability of this approach.

## Methods

### Clinical Specimens

Clinical specimens which included vaginal secretions (220 specimens), leucorrhoea (30 specimens), cauliflower excrescence warts (200 specimens), and other tissues (10 specimens)were collected from Hangzhou First People’s Hospital, China. The patients(210 M, 250F) aged 20 to 50 years.Informed written consent to utilize their specimens for this study was obtained from the patients (Approved by the Ethics Review Committee of Hangzhou First People’s Hospital). After pre-treatment, all of the specimens were subject to DNA extraction, to detection of HPV genotype by HPV GA test (Hybribio Ltd., Hong Kong), and then to quantitative detection of viral loads (only HPV 6, 11, 16 and 18) by fluorescence quantitative PCR (Da’an reagent, Guangzhou Da’an Diagnostic Co., Ltd., China). The positive samples with known concentrations were conserved at –80 °C for future use.

### Construction of Plasmids Containing HPV Genes

We used PCR to gain anticipated size of DNA fragments for each HPV target gene (Table 1) and then ligated to PMD-19T plasmids at 16°C overnight. The ligated products were transformed into E. coli DH5α and plated on LB plates containing l00 mg/L ampicillin. Plasmid DNA was extracted from bacterial culture using an extraction kit (TaKaRa reagent). The plasmid insert DNA sequences (Supporting Information S1) were confirmed by Sanger sequencing (Invitrogen Biotechnology Shanghai, China). The plasmid DNA samples from positive clones were quantified by UV spectrophotometry. Plasmids were diluted to 10^10^ copies/µl in TE (pH 8.0) buffer for storage at –20°C.

**Table 1:** Primers and probes of four types of HPV for AllGlo quadruple quantitative PCR.

Gene ID	Targetgene	Primers	Primer sequences	Probe	Product Length (Bp)
FM897165.1	L1	HPV6FHPV6R	GTTATCGCCTCCCCCAAAT ATCTGGCTTTTCCTTTTCAGG	URA-CCATTACCTGTCAAAAGCCCAC-URA	103
AF217526.1	L1	HPV11FHPV11R	TTGCGAAAGGAACAAATGTTT GGAAGACACCAATGAGCCACT	MAR-TGGGGGAACCTGTGCC-MAR	159
EU869318.1	E6	HPV16FHPV16R	AGGACCCACAGGAGCGAC AGTCATATACCTCACGTCGCAGT	NEP-ATGCACAGAGCTGCAAACAA-NEP	126
EU834744.1	L1	HPV18FHPV18R	GGTTCAGGCTGGATTGCG TACACGCACACGCTTGGC	JUP-TCGCAAACGTTCTGCTCC-JUP	100

URA, MAR, NEP and JUP are different AllGlo probe fluorochromes, URA is red fluorochromes, MAR is green fluorochromes, NEP is orange fluorochromes, JUP is yellow fluorochrome. By BLAST sequence comparison, the specificity of primers were depermined.

### Extraction of the DNA Template from Clinical Specimens

Secretion swab specimens were dissolved in normal saline. Tissues and cauliflower excrescence warts were infiltrated in 4% sodium hydroxide solution and comminuted with metal bar. The dissolved samples were oscillated and resuspended for 1 min, followed by centrifuging for 5 min (4 °C, 12,000×g/min). The supernatant was discarded, and the sediment was resuspended in 50 µl of DNA extraction solution (Guangzhou Da’an Diagnostic Co., Ltd., China). After heating at 100 °C for 10 min, the sample was centrifuged for 5 min (4 °C, 12,000×g/min). The supernatant was saved for qPCR analyses.

### AllGlo Probe Quadruplex Quantitative PCR

#### Primer and probe design

Conserved regions sequences of the four HPV type-specific genes were obtained from Gene Bank. After sequence comparison by CLUSTRALW software, the most conservative regions were used to design the primers and probes with Primer Premiers 5.0. By BLAST sequence comparison, the primers and probes with the best specificity were selected. The primers and AllGlo probes used in the multiplex qPCR were synthesized by Chaoshi Bio-company (Shanghai, China). To ensure the specificity and sensitivity of the multiplex qPCR, all primers and probes were designed to have similar Tm (Table 1). To enhance the hybridization specificity of each probe to its cognate target sequence, each probe was designed to have at least nine mismatched base pairs with its noncognate targets sequence.

#### Determination of the specificity and the sensitivity of the primer and the probe

To determine the specificity and the sensitivity of the primer and the probe, the uniplex fluorescence quantitative PCR experiments were first carried out on a Qiagen Rotor Gene 6000 fluorescence quantitative PCR instrument, which can simultaneously detect five wavelengths. Parameter variables such as cycle temperatures, concentration of primers and probe, the detection limit and replicating steps were all optimized. For every reactions, each 25-µl PCR reaction contained 11 µl of deionized water,10 µl of 2 × qPCR Mix (Chaoshi Bio-company, Shanghai, China), 1 µl individual HPV plasmid DNA template(from 10^2^ copies/µl to10^5^ copies/µl), 2 µl primer (1 µl forward primer, 1 µl reverse primer)and 1 µl probe (four primer/probe combinations and concentrations: 400 nM/400 nM, 400 nM/200 nM, 200 nM/200 nM, or 200 nM/100 nM). Each PCR reaction was performed in triplicate. A blank plasmid without an HPV DNA insert and other HPV types DNA (genotypes identified through HPV GA test and Sanger sequencing,except HPV genotypes 6, 11, 16, 18, such as HPV genotypes 32, 39, 52, 58 etc.) was used as a negative control, and deionized water was used as the no-DNA blank control. The cycling conditions were a 5-min incubation at 95°C followed by 45 temperature cycles moving between 95°C for 10 s and 52°C for 20 s (During the cycling phase, the optimization annealing temperatures were varied from 52 to 66°C in single-degree increments). The specificity and sensitivity of primers and probes were determined according to the lowest Ct value, the highest gain in fluorescence and detection limit of the uniplex fluorescence qPCR, and the quadruplex qPCR described in the following sections referred to the parameters of the uniplex fluorescence qPCR.

#### Optimization of quadruplex quantitative PCR

Thanks to the specificity of the primer and the probe we selected here, we found we could take conditions such as the primer/probe combinations form uniplex assay to multiplex directly. Then we optimized annealing temperature (from 52°C−60°C) and determined the compromise annealing temperature used for multiplex detection was 58?.

Quadruplex quantitative PCR was performed in the same manner as the uniplex qPCR assay described above, PCR mixtures contained 10 µl of 2 × qPCR Mix, 1 µl individual HPV plasmid DNA temple (from 10^2^ copies/µl to10^5^ copies/µl), 8 µl mixture of all four sets of primers and 4 µl mixture of all four sets of probes (four primer/probe combinations and concentrations: 400 nM/400 nM, 400 nM/200 nM, 200 nM/200 nM, or 200 nM/100 nM), 2 µl of deionized water. Other parameter such as cycle temperatures, annealing temperatures and replicating steps were chosen from the uniplex qPCR. Each PCR reaction was performed in triplicate. The negative and blank control test of quadruple qPCR were performed in the same manner as the uniplex qPCR.

#### Determination of the specificity and sensitivity of quadruplex quantitative PCR

Four individual HPV plasmid DNA stocks and one mixed stock that comprised all four types HPV plasmids DNA were made with concentrations ranging from 1.0 × 10^1^ copies/µl to 1.0 × 10^5^ copies/µl. A 1-µl sample of the stock was used as the template for quadruplex fluorescence quantitative PCR. A blank plasmid without an HPV DNA insert and other HPV types DNA (except HPV genotypes 6, 11, 16, 18, such as HPV genotypes 32, 39, 52, 58 etc.) was used as a negative control, and deionized water was used as the no-DNA blank control. Fluorescence qPCR test was carried out in triplicate to (1) determine the minimum detection limit of this method, (2) determine the detection sensitivity and specificity of fluorescence quantitative PCR for the four types of HPV and (3) construct standard curves.

#### Standard curve for quadruplex quantitative PCR

Each quantitative PCR reaction, which was used to construct a standard curve, contained mixture of the four types of HPV plasmids (The concentration of each HPV types was equal) at final concentrations (copies/µl) of 10^3^, 10^4^, 10^5^, 10^6^, and 10^7^, as well as primers and probe at optimized concentrations and combinations. The qPCR assay were performed in duplicate, and a standard curve was constructed by the instrument’s software.

#### The replicability test of quadruplex quantitative PCR

Five aliquots plasmids DNA were made from the mixed stock of four types HPV plasmids and stored at –20 °C, The selected concentrations of each HPV type were 10^2^, 10^4^, 10^6^,and 10^8^(copies/test). Five side-by-side quadruplex quantitative PCR assay were carried out using the aliquots plasmids DNA in triplicate to determine the mean Ct value. The CV% between each aliquot was calculated to determine the replicability of our method.

#### Detection of clinical specimens

270 clinical samples conserved at –80 °C whose HPV genotype and viral loads (only HPV6, 11, 16 and 18) had been previously determined were divided into nine groups (Table 2), each group contained 30 samples. DNA was extracted from these samples with a Da’an extraction kit (Guangzhou Da’an Diagnostic Co. Ltd., Guangzhou, China.). The extracted DNA was divided into five aliquots. Two aliquots were used for the detection of HPV6–11 and HPV16–18 mixed types by TaqMan uniplex probe fluorescence quantitative PCR (Guangzhou Da’an Diagnostic Co., Ltd., China). The third aliquot was used for the detection of HPV 6, 11, 16 and 18 with AllGlo probe quadruplex fluorescence qPCR. The fourth aliquot was used for the HPV genotype detection with HPV GA test again. The remaining aliquot was conserved at –80 °C to further verify the specificity of the quadruplex fluorescence qPCR method, and the same amount of DNA template was used in each experiment. The result was determined only when all parallel detection results were consistent. Finally, the results obtained by the quadruplex qPCR were compared with the results obtained with the uniplex TaqMan qPCR and HPV GA test.

**Table 2:** Comparative results of HPV GA test, TaqMan uniplex qPCR and AllGlo quadruplex qPCR.

HPV TYPE	HPV GA test					TaqManuniplex qPCR		AllGlo quadruplexqPCR			
	6	11	16	18	others	6–11	16–18	6	11	16	18
6	28	0	0	0	0	28	0	29	0	0	0
11	0	28	0	0	0	26	0	0	28	0	0
16	0	0	29	0	0	0	27	0	0	28	0
18	0	0	0	28	0	0	26	0	0	0	28
6+11	28	29	0	0	0	28	0	28	28	0	0
16+18	0	0	27	28	0	0	27	0	0	27	28
6+11+16	26	27	27	0	0	27	27	27	27	26	0
6+11+16+18	27	27	27	28	0	27	28	28	28	27	29
Others^#^	0	0	0	0	30	0	0	0	0	0	0

The agreement rate between AllGlo quadruplex fluorescence quantitative PCR, HPV GA test and TaqMan uniplex qPCR was 100%. Single-tube AllGlo probe quadruplex fluorescence quantitative PCR could simultaneously type HPV 6, 11, 16, and 18 and quantitate the viral load of each HPV at the same time. Compared with the HPV GA test and TaqMan uniplex qPCR method, the AllGlo quadruplex qPCR method enjoys a high sensitivity and a wide linear range. For the same sample, the positive rate and accuracy of the AllGlo quadruplex qPCR method was higher than those of the HPV GA test and TaqMan uniplex qPCR method. ^#^Others:other HPV type,including HPV31,33,35,39,42,43,44,45,51,52,53,56,58,59,66,68,CP8304.

## Results

### Concentrations and Combinations of the Primer and the Probe, Annealing Temperature

Titrations of primer/probe combinations gave a positive result in uniplex qPCR and Quadruplex qPCR. The 400/200 combination had the highest amplification efficiency and the lowest Ct value (Table 3). The optimum annealing temperatures (°C) for HPV 6, 11, 16 and 18 amplification were: 60, 58, 58 and 58, respectively. In quadruplex fluorescence quantitative PCR, the compromise annealing temperature used for multiplex detection was 58°C, the primer/probe combinations was 400/200 (nM).

**Table 3:** Comparison of sensitivity and detection limit between TaqMan uniplex qPCR and AllGlo Quadruplex qPCR.

HPVTYPE	10^1^ copies/test		10^2^ copies/test		10^3^ copies/test		10^4^ copies/test		10^5^ copies/test	
	uniplex qPCR(Ct*)	Quadruplex qPCR (Ct)	uniplexqPCR(Ct)	QuadruplexqPCR (Ct)	uniplex qPCR(Ct)	QuadruplexqPCR (Ct)	uniplex qPCR (Ct)	Quadruplex qPCR (Ct)	uniplex qPCR (Ct)	Quadruplex qPCR (Ct)
6	0	0	31.1±2.2	31.8±2.3	27.2±1.8	28.1±1.7	24.8±1.6	25.1±1.5	21.8±1.2	21.6±1.3
11	33.9±1.6	34.5±1.8	29.6±1.3	29.8±1.2	25.7±1.1	26.1±1.3	22.4±0.8	22.8±1.0	18.9±0.6	18.5±0.7
16	0	0	30.1±1.6	31.1±1.8	26.5±1.5	26.9±1.7	22.9±1.1	22.7±1.2	19.8±1.0	19.9±1.2
18	33.0±1.9	33.5±1.6	29.9±1.5	30.1±1.6	26.1±1.2	26.2±1.2	22.6±0.9	22.1±1.0	19.3±0.8	18.9±0.9
Others#	0	0	0	0	0	0	0	0	0	0
Negative	0	0	0	0	0	0	0	0	0	0

# Others:other HPV type,including HPV31,33,35,39,42,43,44,45,51,52,53,56,58,59,66,68,CP8304.

* cycle threshold (Ct) value.

### Specificity, Sensitivity and Replicability of Quadruplex Fluorescence Quantitative PCR

**Figure 2:**
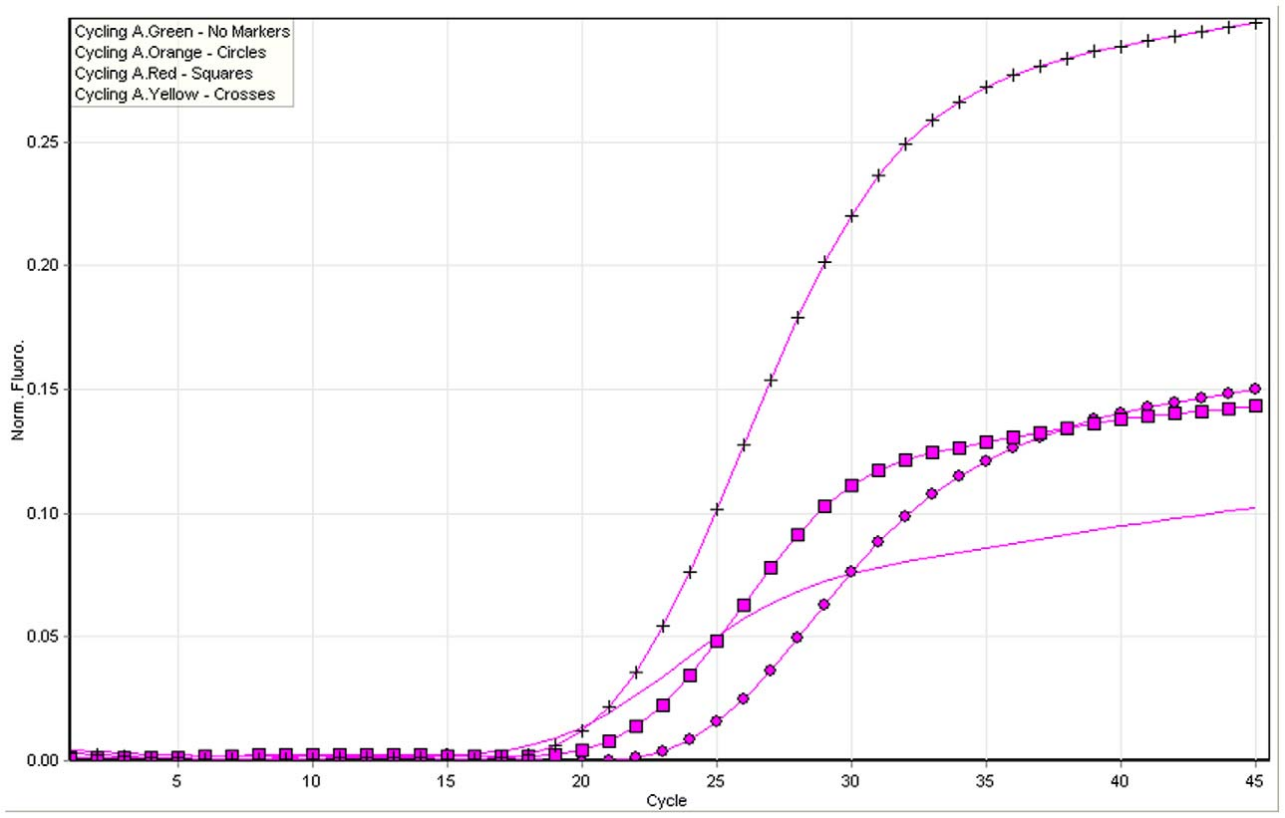
Specificity test of AllGlo probe quadruplex fluorescence quantitative PCR method. The concentration of each four HPV plasmid mixture (HPV 6, 11, 16, and 18) was 10^5^copies/test.HPV6-red (URA),HPV11-no markers (MAR),HPV11-circles (NEP), HPV18-crosses (JUP).

All of the four HPV types in quadruplex qPCR were detected positively, while the other type of HPV were negative in the same detection system (Figure 2). The sensitivities (copies/test) for HPV 6, 11, 16, and 18 were 10^1^, 10^2^, 10^1^, and 10^2^ respectively (Table 4).The detection result of quadruplex qPCR is consistent with the uniplex qPCR(P>0.05). The coefficients of variation (CVs) of the five parallel tests for the four types of HPV plasmids were all below 5% (Table 4).

**Table 4:** Results of replicability test of AllGlo quadruplex qPCR.

Concentration*	HPV6		HPV11		HPV16		HPV18	
	Intra#	Inter 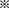	Intra	Inter	Intra	Inter	Intra	Inter
10^2^	<1.1	1.43	<1.16	0.90	<1.31	1.00	<1.39	1.02
10^4^	<0.86	0.78	<1.25	0.95	<1.27	0.96	<1.46	1.23
10^6^	<1.21	0.80	<1.07	0.79	<1.21	0.99	<1.26	0.86
10^8^	<1.17	0.97	<0.92	0.72	<1.96	1.12	<1.16	0.95

Five side-by-side AllGlo quadruplex quantitative PCR assay were carried out using the DNA aliquots in triplicate to determine the mean CT value, and the mean CT value was used for statistics of coefficient of variation (%) for four HPV types.

* copies/test.

# Intra-assay coefficient variation.

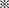
Inter-assay coefficient variation.

### Preparation of a Standard Curve

Five concentrations of plasmids of each type (10^3^, 10^4^, 10^5^, 10^6^, and 10^7^ copies/test) were used to construct a standard curve with the AllGlo quadruplex fluorescence quantitative PCR system. The mean amplification efficiencies for HPV 6, 11, 16, and 18 were 0.97, 1.10, 0.93 and 1.20, respectively, and the mean correlation coefficient (r^2^) of each standard curve was above 0.99.

### Results of Clinical Specimens Detection

The results obtained with AllGlo quadruplex fluorescence qPCR, HPV GA test and TaqMan uniplex qPCR method were summarized in Table 2.The agreement rate between AllGlo quadruplex fluorescence qPCR, HPV GA test and TaqMan uniplex qPCR was 100%. Single-tube AllGlo probe quadruplex fluorescence qPCR could simultaneously type HPV 6, 11, 16, and 18 and quantitatively detect the viral load of each HPV at the same time. The detection sensitivity of AllGlo probe quadruplex fluorescence qPCR was higher than that of HPV GA test and TaqMan uniplex qPCR.

## Discussion

The fluorescence PCR technique is becoming increasingly important in the diagnosis of human diseases, such as influenza virus, avian flu virus [30,31], cytomegalovirus (CMV) [32], and severe acute respiratory syndrome (SARS) virus [33]. Fluorescence PCR has advantages over conventional PCR [34,35].The former generates an amplification kinetics curve, which directly reflects the dynamic changes of the PCR process in real time. With the development of molecular biology and molecular marker techniques, multiplex fluorescence quantitative PCR technology is becoming more widely used. Multiplex PCR not only significantly reduces the workload but also increases the accuracy and comparability of the experiment. Methodologically, the multiplex fluorescence quantitative PCR technique is also suitable for HPV viral load and genotyping detection, especially for detecting a large number of samples simultaneously.

Quantitative PCR has been developed based on conventional PCR techniques. The quantitation of DNA or RNA templates is achieved by determining how many cycles are needed to reach a certain level of fluorescence. Currently used probes in fluorescence PCR include TaqMan probes, FRET probes, molecular signal probes, new fluorescent double-stranded replacement probes [36,37] and AllGlo probes. FRET probes are not commonly used in real-time monitoring but are used in melting-point analysis and genotyping after the completion of PCR amplification [38]. It is difficult to achieve the specificity in genotyping with conventional TaqMan probes; MGB was reported to enhance the specificity. However, MGB increases the cost and the difficulties in probe design and synthesis [39]. Molecular signal probes have complicated designs when used in genotyping, and they are also inefficient in single-base discrimination [40]. Currently, the most widely used probe in the clinical laboratory is TaqMan. However, it is difficult to design TaqMan experiments to detect multiple targets simultaneously, and this seriously limits the application of real-time PCR in genotyping [41,42].

The AllGlo probe is the latest generation of quantitative fluorescent probes invented by AlleLogic Biosciences Corporation. AllGlo probes use two identical fluorophores that are specially selected in such a way that the two fluorophores are mutually quenched when both are conjugated to the probe oligonucleotide. Both fluorophores become dequenched when the probe is cleaved. This study, the AllGlo probe quadruplex quantitative PCR was performed to simultaneously detect and differentiate human papillomavirus genotypes 6, 11, 16 and 18. Samples of four types of HPV (HPV 6, 11, 16, and 18) were PCR-amplified and sequence-confirmed in order to ensure the reliability of the fluorescence qPCR. Plasmids containing the target gene were cloned by T-A cloning for the preparation of standard DNA stock. By optimizing the concentrations and combinations of primers and probes in AllGlo quadruplex quantitative PCR, we finally resolved the issue of mutual interference among primers in multiplex PCR and established a single-tube quadruplex fluorescence quantitative PCR technique based on the AllGlo probe. This new approach allows simultaneous detection and typing of four HPV types in a single tube. The r^2^ values of all of the standard curves were larger than 0.99, and the amplification efficiency was between 0.91 and 1.23, which meets the requirement for fluorescence quantitative PCR standard curves [43,44]. The agreement rate between AllGlo quadruplex fluorescence qPCR, HPV GA test and TaqMan uniplex qPCR was 100%. Single-tube AllGlo probe quadruplex fluorescence qPCR could simultaneously type HPV 6, 11, 16, and 18 and quantitatively detect the viral load of each HPV at the same time. Compared with the HPV GA test and TaqMan uniplex qPCR method, the AllGlo quadruplex qPCR method enjoys a high sensitivity and a wide linear range. In our experiment, the sensitivity of quadruplex fluorescence quantitative PCR can reach 10 to100 copies/test, while the sensitivity of HPV GA test and TaqMan uniplex qPCR method was above 100 copies/test. For the same sample, the positive rate and accuracy of the AllGlo quadruplex qPCR method was higher than those of the HPV GA test and TaqMan uniplex qPCR method. In addition, AllGlo probe quadruplex fluorescence quantitative PCR also has the advantages of relatively high throughput, time savings, simple operation, and lower cost, which are key factors that are needed in order to be qualified for clinical applications. The repeatability, sensitivity and specificity were high enough to be qualified for both qualitative and quantitative detection in clinical settings.

### Conclusion

AllGlo quadruplex quantitative PCR has the advantages of relatively high throughput, good reproducibility, high sensitivity, high specificity, and a wide linear range of detection. The convenient single-tube format makes this assay a powerful tool for the study of mixed infections caused by multiple pathogens, viral typing and viral load quantification,meanwhile, this method could be used in single nucleotide polymorphism (SNP) and mutation analysis, prenatal diagnosis and genetic disease screening, drug efficacy analysis, tumor gene expression detection, and so on. The assay will facilitate molecular epidemiological studies and drug development.

## Supporting Information

Supporting Information S1 **The DNA target sequences of HPV (HPV 6, 11, 16, 18) inserted in plasmid was confirmed by Sanger sequencing.**
(DOC)
