# Supplementary material for: Simultaneous Detection and Differentiation of Human Papillomavirus Genotypes 6, 11, 16 and 18 by AllGlo Quadruplex Quantitative PCR
Source: PLoS One. 2012 Nov 9;7(11):e48972. doi: 10.1371/journal.pone.0048972 (PMC3494670; doi:10.1371/journal.pone.0048972)
Supplement: Supporting Information S1 — The DNA target sequences of HPV (HPV 6, 11, 16, 18) inserted in plasmid was confirmed by Sanger sequencing. (DOC) [file pone.0048972.s001.doc]

**Supporting Information File**

**S1 The DNA target sequences of HPV (HPV 6, 11, 16, 18) inserted in plasmid was confirmed by Sanger sequencing.**

**HPV6 target sequences (103bp)**

ATCTGGCTTTTCCTTTTCAGGAGTGGGCTTTTGACAGGTAATGGCCTGTGACTGCACATACCTATAGGTATCTTCTAATGTACCATTTGGGGGAGGCGATAAC

**HPV11 target sequences (159bp)**

GGAAGACACCAATGAGCCACTAGGTGTATGTACATAAATACTACTAGCTACAGATGATCTATTATTACCCCCTTTTACTAACAGGTCATCAGGCACAGGTTCCCCCACAGTACCGGCCCTATTAAAAAAGTGTCTAGCAAACATTTGTTCCTTTCGCAA

**HPV16 target sequences (126bp)**

AGTCATATACCTCACGTCGCAGTAACTGTTGCTTGCAGTACACACATTCTAATATTATCTCATGTATAGTTGTTTGCAGCTCTGTGCATAACTGTGGTAACTTTCTGGGTCGCTCCTGTGGGTCCT

**HPV18 target sequences (100bp)**

TACACGCACACGCTTGGCAGGTTTAGAAGACGTAGTGGCAGATGGAGCAGAACGTTTGCGAGGGCCTATGGTGGGCTTGCGACGCAATCCAGCCTGAACC
